# Bioinformatic analysis of circular RNA expression profiles in a rat lumbosacral spinal root avulsion model

**DOI:** 10.3389/fgene.2022.920493

**Published:** 2022-08-12

**Authors:** Zhibin Zhou, Jun Ma, Jiao Cai, Aimin Chen, Lei Zhu

**Affiliations:** ^1^ Department of Orthopaedics, General Hospital of Northern Theater Command, Shenyang, Liaoning, China; ^2^ Department of Orthopaedics, Naval Medical Center of PLA, Naval Medical University, Shanghai, China; ^3^ Department of Medical Administration, Second Affiliated Hospital of Naval Medical University, Shanghai, China; ^4^ Department of Orthopaedics, Second Affiliated Hospital of Naval Medical University, Shanghai, China

**Keywords:** lumbosacral nerve root avulsion, neuron, circular RNA, RNA sequencing, bioinformatics

## Abstract

Lumbosacral spinal root avulsion (LSRA) is a severe nerve injury that results in devastating dysfunction in the lower limb. Circular ribonucleic acids (circRNAs) have been reported to be implicated in a variety of diseases. However, the role of circRNAs in LSRA remains unclear. Here, we performed RNA sequencing (RNA-seq) to determine circRNA expression profiles in a rat LSRA model and further investigated their potential functions and the underlying mechanisms by bioinformatic analyses and *in vitro* experiments. In all, 1708 circRNAs were found to be differentially expressed in spinal cord tissues after LSRA (|fold change| ≥ 2 and *p* < 0.05), with 591 up-regulated 1117 down-regulated. Meanwhile, 2263 mRNAs were also indentified to be differentially expressed, of which 1471 were upregulated and 792 were downregulated. Eight randomly selected circRNAs and mRNA were successfully verified to be consistent the RNA-seq results by quantitative real-time polymerase chain reaction. Functional analyses based on gene ontology and Kyoto Encyclopedia of Genes and Genomes predicted the potential roles of differentially expressed circRNAs and mRNAs in LSRA, and circRNA/miRNA/mRNA interaction networks revealed that circRNA_7025, a down-regulated circRNA in LSRA, was targeted by two neuronal apoptosis-related miRNAs, rno-miR-1224 and rno-miR-326-5p. Further *in vitro* experiments revealed that circRNA_7025 protected against oxygen-glucose deprivation induced neuronal apoptosis *via* the circRNA_7025/miR-1224/miR-326-5p axis. In summary, our results revealed circRNA expression profiles and their potential functions in LSRA. These findings improve our understanding of the pathogenic mechanisms involved in LSRA and might enable us to identify new molecular targets for LSRA.

## Introduction

Lumbosacral spinal root avulsion (LSRA) mainly occurs during high-speed traffic accidents, resulting in permanent loss of sensation and motor function in the lower limb (Sidhu and Dhillon, 1991; Chin and Chew, 1997). LSRA is not a common clinical entity, and only 35 cases were reported since the first description in 1955 (Chin and Chew, 1997). LSRA is not often reported in the literature because of low incidence and difficulty in reaching a precise diagnosis, as well as the complicated treating methods (Barnett and Connolly, 1975). For the above reasons, the incidence is probably underestimated. Currently, the operative methods for patients with LSRA are limited and consist mainly of avulsed nerve root reimplantation and S1 nerve root transfer (Gu et al., 2004; Zhu et al., 2015). Despite the great progress in neurosurgical repair, the functional recovery of LSRA is far from optimal because of limited understanding of the pathogenic mechanism (Lang et al., 2004). Although considerable effort has been made to reveal the pathological process of LSRA, and our previous studies found that the death of neurons regulated by two canonical cell death pathways, apoptosis and autophagy, is one of the key factors that restricting the repair effects (Jiang et al., 2014; Zhu et al., 2016), the exact molecular mechanism of LSRA still remains to be elucidated. Therefore, for the development of new treatment strategies, a comprehensive understanding of the pathophysiology and mechanism for LSRA is need.

Circular ribonucleic acids (circRNAs) are a new class of noncoding RNAs characterized by covalently closed loop structures (Memczak et al., 2013). It has been shown that circRNAs are widely expressed in eukaryotic cells and have important roles such as function as microRNA (miRNA) sponges (Memczak et al., 2013), binding with proteins (Li et al., 2015), and translating into proteins (Legnini et al., 2017). Presently, accumulating evidence indicates that circRNAs are involved in a various of diseases, such as diabetes mellitus (He et al., 2020), osteoarthritis (Zhou et al., 2019a), acute ischemic stroke (Zuo et al., 2020) and malignant tumors (Tang et al., 2021). Additionally, previous studies reported that traumatic brain injury results in significant changes in circRNA expression profiles and some of the differentially expressed circRNAs may have important regulatory functions (Xie et al., 2018; Zhao et al., 2018). We also found that differentially expressed circRNAs were induced following spinal cord injury and they might be involved in both the primary and secondary stage (Zhou et al., 2019b). However, the role of circRNAs in LSRA remains unclear.

To investigate weather circRNAs participate in the pathological process of LSRA, and preliminarily predict their potential functions, we established a rat LSRA model to explore the expression profiles of circRNAs and their potential functions by combining RNA sequencing (RNA-seq), qRT-PCR, and bioinformatics. Mechanistically, we further found that circRNA_7025 might play an anti-apoptotic role in LSRA by targeting rno-miR-1224 and rno-miR-326-5p. Thus, our study advances our understanding of the pathogenic mechanisms and reveals new molecular targets for the treatment of LSRA.

## Materials and methods

### Animals and lumbosacral spinal root avulsion model

Adult male Sprague-Dawley (SD) rats (weighted 200–220 g, aged 8 weeks) were purchased from Shanghai SLAC Laboratory Animal Co., Ltd. and used for establishment of the LSRA model as previously described (Zhu et al., 2016). In short, rats were anesthetized with intraperitoneal injection of pentobarbital sodium (1%, 40 mg/kg; Tc-P8411, Merck, Germany). The right L4–L6 nerve roots were exposed and avulsed with a small homemade instrument for rats in the LSRA group. For rats in the Sham group, the right L4–L6 nerve roots were only exposed but not avulsed. In all, a total of 38 rats were randomly assigned to the following groups: for RNA sequencing (RNA-seq) experiment, Sham group (*n* = 9), LSRA group (*n* = 9); for Quantitative real-time polymerase chain reaction (qRT-PCR) verification experiment, Sham group (*n* = 10), LSRA group (*n* = 10). The rats were sacrificed 1 day after surgery and the corresponding spinal cord tissues of L4–L6 segments were obtained for further experiments. All experiments were performed in accordance with the guidelines of the Animal Ethics Committee of Second Affiliated Hospital of Naval Medical University (2022SLYS3).

### RNA isolation and RNA sequencing

After anesthesia, the rats were sacrificed and the corresponding spinal cord tissues of L4–L6 segments were harvested for RNA isolation and RNA-seq. The spinal cord tissues, including the avulsion epicenter, were harvested, frozen, and stored in liquid nitrogen. For RNA-seq, the spinal cord tissues of three rats in the same group were mixed into one sample, so each group had three samples (*n* = 3 rats/sample, *n* = 3 samples/group). High-throughput RNA-seq was performed by OE Biotech Co., Ltd. (Shanghai, China). Briefly, total RNA was extracted using the mirVana miRNA Isolation Kit (Ambion-1561, Ambion, TX, United States) following the manufacturer’s protocol. RNA integrity was evaluated using the Agilent 2100 Bioanalyzer (Agilent Technologies, CA, United States). The samples with RNA Integrity Number (RIN) ≥ 7 were subjected to the subsequent analysis. The libraries were constructed using TruSeq Stranded Total RNA with Ribo-Zero Gold (RS-122-2301, Illumina, CA, United States) according to the manufacturer’s instructions. Then these libraries were sequenced on the ilumina Novaseq 6000 platform (Illumina, CA, United States) and 150 bp paired-end reads were generated.

### Data preprocessing and RNA quantification

Raw reads generated from RNA-seq were further processed using Trimmomatic (Bolger et al., 2014) to obtain high-quality clean reads. Then the clean reads were mapped to the rat reference genome (Rnor_6.0) using HISAT2 (Kim et al., 2015), and quantification of mRNAs were performed by calculating the fragments per kb per million reads (FPKM) value using Cufflinks (Trapnell et al., 2010) and counts value (the number of reads for each gene in each sample) using HTSeq-count (Anders et al., 2015). Differential expression analysis was performed using the DESeq2 (Love et al., 2014), and |fold change| ≥ 2 and *p* < 0.05 was set as the threshold for significantly differential expression gene.

For circRNA identification and quantification, we used BWA-MEM software (Li, 2013) to align the sequencing reads of each sample with the rat reference genome to obtain sequence alignment/map (SAM) files, and the paired chiastic clipping signals in SAM files were scanned using the CIRI2 software (Gao et al., 2018) and circRNA sequences were predicted based on junction reads and GT-AG cleavage signals. Then the expression of circRNAs were calculated using SRPM (spliced reads per million mapping) (Li et al., 2014), and the differential expression analysis was also using the DESeq2 with the same criteria of genes.

### Quantitative real-time polymerase chain reaction analysis

We conducted qRT-PCR to verify the expression levels of eight randomly selected circRNAs and mRNAs. Total RNA was extracted from spinal cord tissues using Trizol reagent (15596026, Invitrogen, CA, United States) according to the manufacturer’s protocol. RNA integrity was evaluated using the NanoDrop 2000 (Thermo Fisher Scientific, MA, United States). Total RNA was reverse transcribed to synthesize first-strand cDNA using a Prime Script RT Reagent Kit (RR047A, TaKaRa, Osaka, Japan) and qRT-PCR analyses were performed using a Roche Applied Science Light Cycler 480II Real-time PCR system (Roche Applied Science, IN, United States) according to the manufacturer’s instructions. Gapdh and U6 were served as internal controls for circRNAs and miRNAs. Data were calculated by the 2^−ΔΔCt^ method. The primers used for qRT-PCR are listed in Supplementary Data S1.

### Bioinformatic analysis

Based on the hypergeometric distribution, Gene Ontology (GO) and Kyoto Encyclopedia of Genes and Genomes (KEGG) pathway enrichment analysis of the parental genes of the differentially expressed circRNAs and differentially expressed mRNAs were performed to screen the significant enriched term using “ClusterProfiler” package (Yu et al., 2012) in R (Version 3.2.0). The significant enrichment term of GO and KEGG pathway were illustrated by the column diagram and bubble diagram, respectively.

CircRNA/miRNA interactions were predicted by using software miranda (Enright et al., 2003), with the parameter as follows: S ≥ 150, ΔG ≤ −30 kcal/mol and demand strict 5′ seed pairing, and circRNA/miRNA interaction enrichment score and *p* valve were calculated based on the hypergeometric test of predicted miRNAs and differentially expressed circRNAs as previous described (Wu et al., 2020). Then the interactions were ranked according to *p* value of the hypergeometric distribution and the top 300 circRNA/miRNA interactions were selected to construct a circRNA/miRNA network using the Cytoscape software (Version 3.9.1) (Shannon et al., 2003). Besides that, targeted mRNAs of rno-miR-1224 and rno-miR-326-5p were predicted by TargetScan (www.targetscan.org/vert_71) and a circRNA/miRNA/mRNA interaction network was also visualized by the Cytoscape software.

### Primary neuron isolation, culture and oxygen and glucose deprivation treatment

Primary spinal neurons were isolated as previously described (Zhu et al., 2016). Briefly, female SD rats (weighted 240–260 g, aged 10–12 weeks, Shanghai SLAC Laboratory Animal Co.,Ltd.) were used to obtain 2-week embryos, and the spinal cord tissues of the embryos were harvested and cut into small pieces for neuron isolation using 0.05% trypsin (25200072, Thermo Fisher Scientific, MA, United States). Then, neurons were diluted to the indicated density and plated onto poly-l-lysine-coated culture plates (Sigma, MO, United States). We cultured the neurons with serum-free neurobasal medium (21103049, Thermo Fisher Scientific, MA, United States) supplemented with 2% B27 supplement (17504044, Thermo Fisher Scientific, MA, United States). For OGD treatment, we used serum-free and glucose-free DMEM (A4192101, Thermo Fisher Scientific, MA, United States) and then placed the cells in a hypoxic incubator chamber (5% CO2, 95% N2; (17504044, Thermo Fisher Scientific, MA, United States) for 4 h. The control cells were incubated in complete medium with normal oxygenation (20% O2, 5% CO2).

### Luciferase reporter assay

We used the luciferase reporter assay to detect the direct binding between circRNA_7025, rno-miR-1224 and rno-miR-326-5p. Wild type and mutant luciferase reporter vectors of circRNA_7025 were constructed by inserting circRNA_7025 or their mutant fragments into pGL3-firefly_luciferase vector. HEK-293T cells were seeded in 96-well plates at a density of 5 × 10^3^ cells/well and cultured for 24 h. Then 500 ng luciferase reporter vectors were mixed with 20 nmol rno-miR-1224 and rno-miR-326-5p mimics or their negative control plasmids, and they were cotransfected into HEK-293T cells using Lipofectamine 2000 (11668030, Thermo Fisher Scientific, MA, United States). After incubating for 48 h, the luciferase activity was analyzed using the dual luciferase reporter system (E1910, Promega, WI, United States) and relative luciferase activity was determined by calculating the ratio between firefly and Renilla luciferase.

### Plasmid construction and transfection

Overexpression vectors of circRNA_7025, rno-miR-1224, rno-miR-326-5p, and their negative control plasmid vectors were designed and constructed. Primary spinal neurons were (2-6 × 10^5^/well) were cultured in 6-well plates for 24 h, and then plasmid vectors were transfected into cultured neurons with Lipofectamine 2000 (11668030, Thermo Fisher Scientific, MA, United States) under the guidance of the manufacturer’s instructions. After incubated for 48 h, neurons were subjected to OGD treatment and then harvested for further experiments.

### Flow cytometry analysis

Neuronal apoptosis was measured using flow cytometry analysis. After transfection of certain vectors for the indicated hours, neurons received OGD treatment and were washed with 4°C PBS and collected with 0.25% trypsin for flow cytometry analysis. Neurons were incubated with 5 μl FITC-Annexin V and 5 μl PI for 15 min according to instructions of the Annexin V-FITC/PI Apoptosis Kit (Servicebio, Wuhan, China) and analyzed using a FACSCalibur flow cytometer (BD Biosciences, NJ, United States).

### Statistical analysis

We performed three independent repeated experiments to obtain data in this study. Data are presented as the mean ± standard deviation. Statistical analyses were performed using Graphpad Prism nine and statistical significance was determined by Student’s t-test or Spearman correlation coefficients test where appropriate. A two-side *p*-value < 0.05 was defined as statistically significant.

## Results

### The expression profiles of Circular ribonucleic acids in lumbosacral spinal root avulsion

**FIGURE 1:**
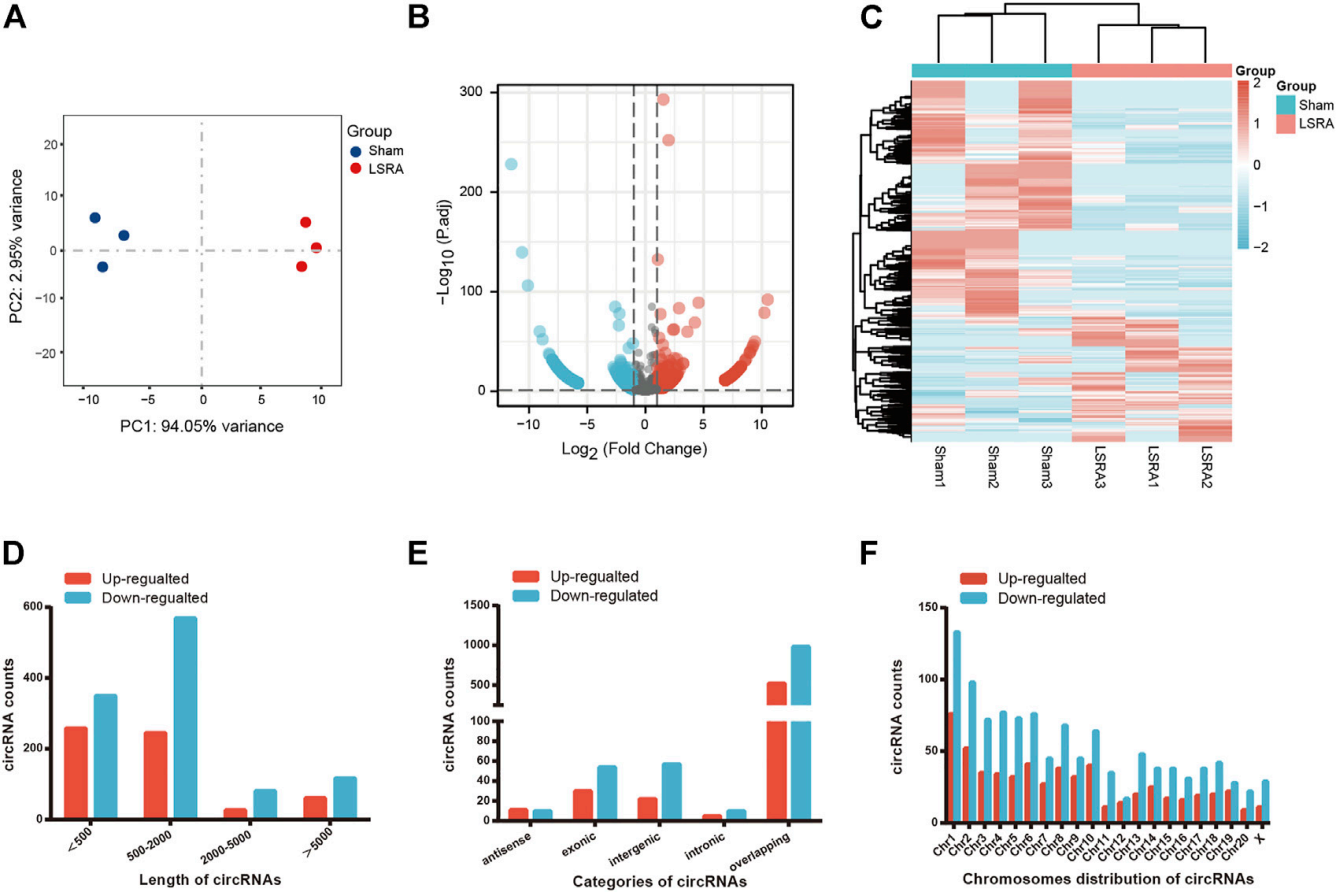
The expression profiles of circRNAs. **(A)** PCA analysis showing distinct clustering of the individual samples of Sham and LSRA groups. **(B)** Volcano plot of circRNA expression (*n* = 3). **(C)** Hierarchical clustering analysis of circRNAs that were markedly differentially expressed (|fold change| ≥ 2 and *p* < 0.05) between the two groups (*n* = 3). **(D)** Length distributions of differentially expressed circRNAs. **(E)** Five categories of circRNAs based on genomic origin. **(F)** Distribution of the differentially expressed circRNAs on the rat chromosomes. circRNAs, circular RNAs; LSRA, lumbosacral spinal root avulsion.

We performed RNA sequencing to screen differentially expressed circRNAs in three pairs of spinal cord tissues from rats in Sham and LSRA groups. Principle component analysis (PCA) showed distinct clustering of the individual samples of Sham and LSRA groups (Figure 1A), indicating the rigor and reproducibility of RNA sequencing data obtained from different samples. Differentially expressed circRNAs were identified by volcano plot filtering (Figure 1B). Furthermore, the differentially expressed circRNAs were transferred to a heatmap to display circRNA expression profiles (Figure 1C).

In all, we found 1708 differentially expressed circRNAs (|fold change| ≥2 and *p* < 0.05), of which 591 circRNAs were upregulated and 1117 circRNAs were downregulated (Supplementary Data S2). Among these differentially expressed circRNAs, approximately 83% of them were <2000 nts in length (Figure 1D). CircRNAs are usually divided into five types: “exonic circRNA” represents a circRNA arising from the exons of a linear transcript; “intronic circRNA” represents a circRNA arising from an intron of a linear transcript; “antisense circRNA” represents a circRNA whose gene locus overlaps with the linear RNA but is transcribed from the opposite strand; “sense overlapping circRNA” represents circRNA originating from the same gene locus as the linear transcript; “intergenic circRNA” represents a circRNA arising from the sequence located in the genomic interval between two genes (Memczak et al., 2013; Zheng et al., 2016). Among them, 21 circRNAs were transcribed from the antisense region, 84 from the exonic regions, 79 from the intergenic regions, 15 from the intronic regions, and 1509 from the overlapping regions (Figure 1E). Figure 1F shows the distribution of differentially expressed circRNAs on the rat chromosomes, and both the upregulated and downregulated circRNAs were transcribed from all chromosomes.

### Gene ontology and kyoto encyclopedia of genes and genomes pathway analyses of differentially expressed circular ribonucleic acids

**FIGURE 2:**
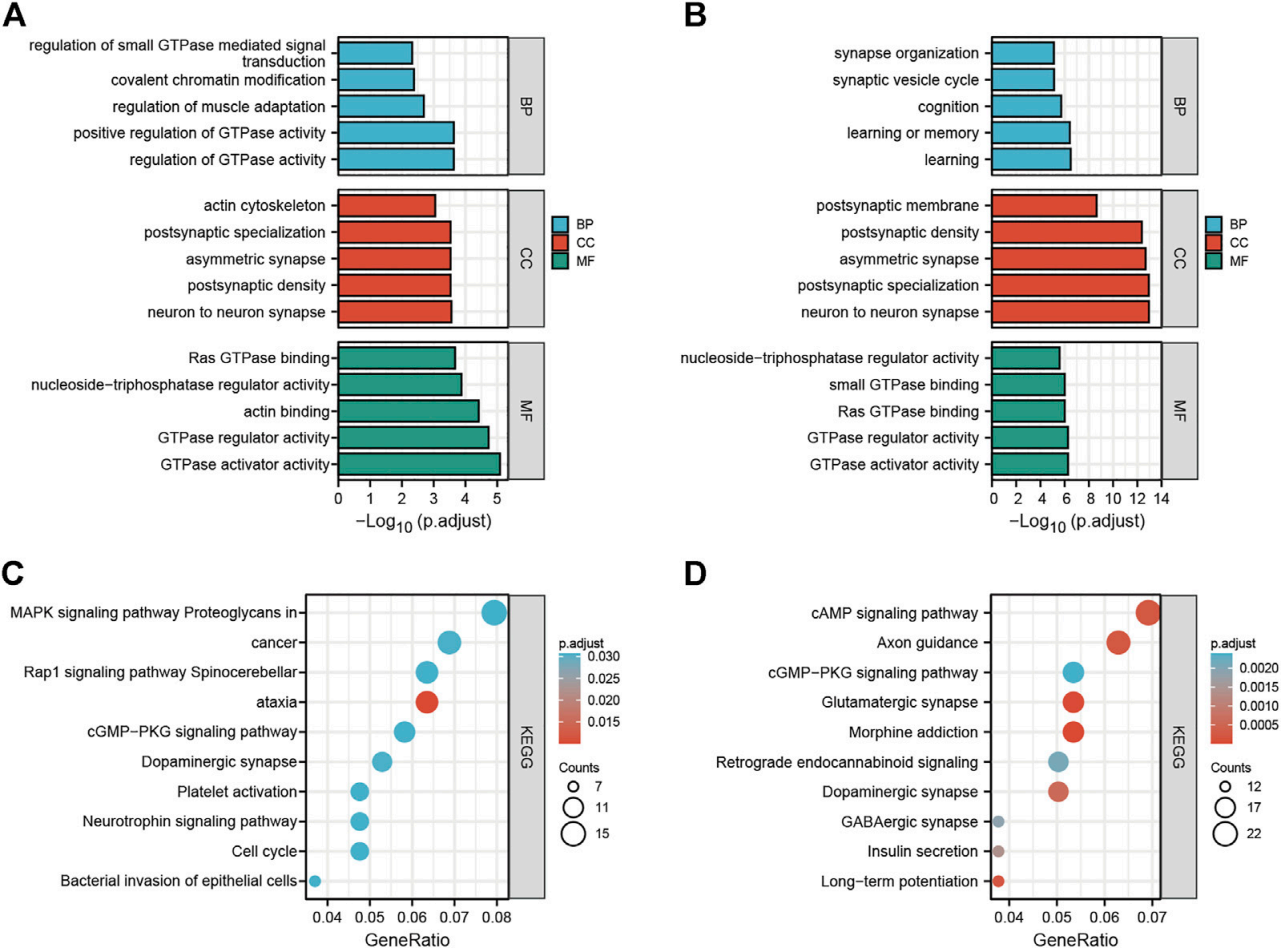
GO and KEGG pathway analyses of differentially expressed circRNAs. **(A)** Top five enriched GO terns for the parental genes of upregulated circRNAs. **(B)** Top five enriched GO terns for the parental genes of downregulated circRNAs. **(C)** Top 10 KEGG terms of parental genes of upregulated circRNAs. **(D)** Top 10 KEGG terms of parental genes of downregulated circRNAs. GO, Gene Ontology; KEGG, Kyoto Encyclopedia of Genes and Genomes; circRNAs, circular RNAs.

To explore the potential functions and possible biological pathways of differentially expressed circRNAs after LSRA, GO analysis and KEGG pathway analysis were performed for the parental genes of the differentially expressed circRNAs (Supplementary Datas S3, S4). GO analyses were performed in the BP, CC and MF domains. The enrichment score [−log10 (*p* value)] was used for the ranking of GO entries, and the top five enriched GO terms in different domains for parental genes are displayed in Figures 2A,B. Notably, the most enriched terms in CC and MF for parental genes of up- and downregulated circRNAs are the same: “neuron to neuron synapse” and “GTPase activator activity”, while the most enriched term in BP for parental genes of upregulated and downregulated circRNAs were “regulation of GTPase activity” and “learning”, respectively.

In the KEGG pathway analysis, the top 10 pathways associated with the parental genes of the upregulated and downregulated circRNAs are shown in Figures 2C,D, respectively. The most enriched and meaningful pathways among these pathways were mainly involved in the “MAPK signaling pathway” and “cAMP signaling pathway”. Besides that, the top 10 up- and down-regulated circRNAs were showed in Supplementary Data S5, and functional annotation of their parental genes were also performed. We found that, among the parental genes of these 20 differentially expressed circRNAs, 11 out of 20 genes were also differentially expressed in LSRA samples (*p* < 0.05), and the most significantly changed parental gene for up- and down-regulated circRNAs are Ttn and Cntn1, respectively. Furthermore, the GO and KEGG analyses results showed distinct functions of the parental genes of these circRNAs, such as the cAMP signaling pathway, the TGF-beta signaling pathway and the cGMP - PKG signaling pathway.

### Identification and functional annotation of differentially expressed messenger RNAs

**FIGURE 3:**
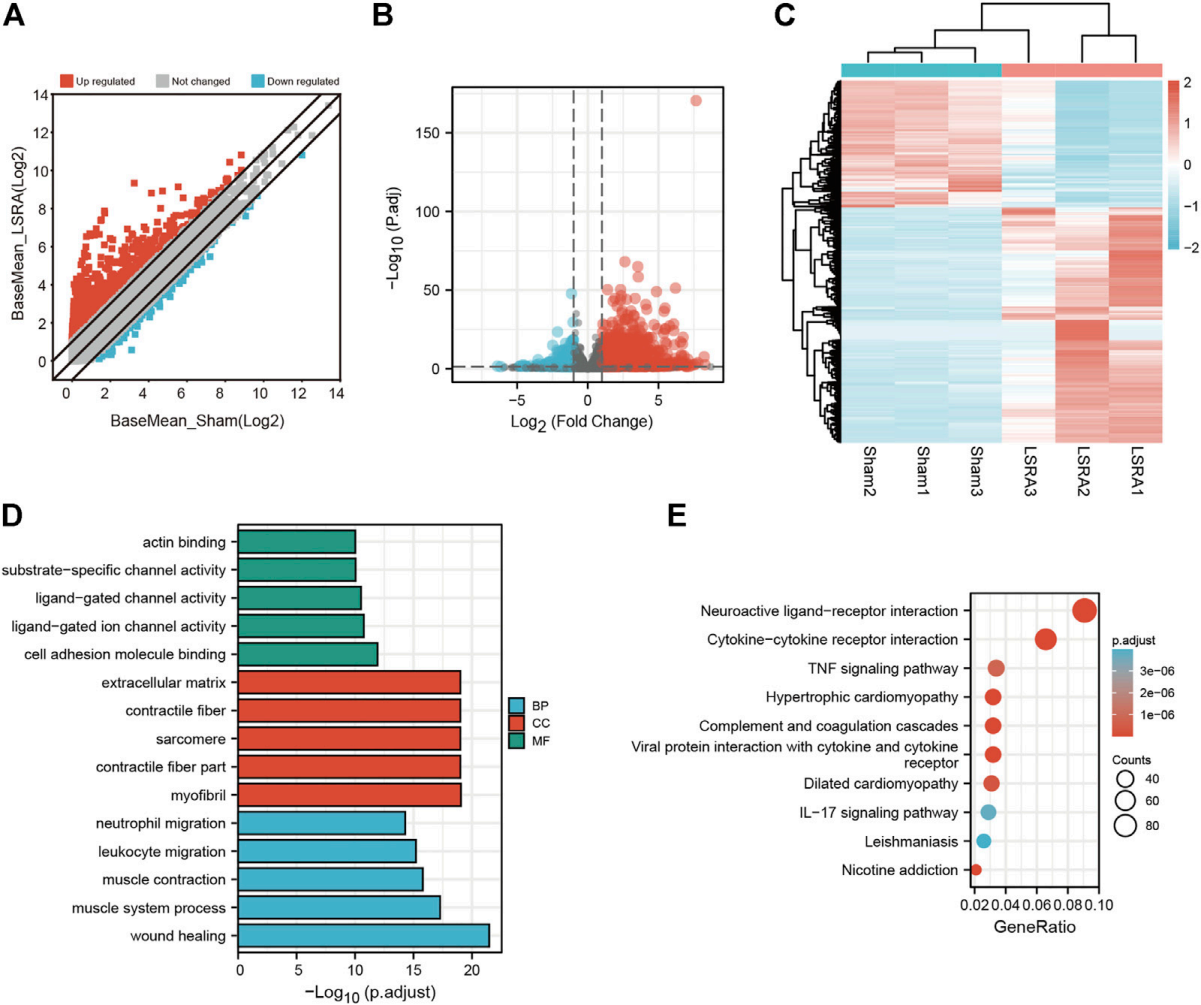
Identification and functional annotation of differentially expressed mRNAs. **(A)** Volcano plot of mRNA expression between the Sham and LSRA groups. **(B)** Scatter plot of mRNA expression between the Sham and LSRA groups. **(C)** Hierarchical clustering analysis of mRNAs that were markedly differentially expressed (|fold change| ≥ 2 and *p* < 0.05). **(D)** Top five enriched GO terms for the differential expressed mRNAs. **(E)** Top 10 enriched KEGG terms of differential expressed mRNAs. mRNAs, messenger RNAs; LSRA, lumbosacral spinal root avulsion; GO, Gene Ontology; KEGG, Kyoto Encyclopedia of Genes and Genomes.

Then the differentially expressed mRNAs were also analyzed. The scatter plot and volcano plot filtering showed differentially expressed mRNAs identified by *p* values and foldchange between Sham and LSRA group (Figures 3A,B). In all, 2263 mRNAs were indentified to be differentially expressed between the two groups with |fold change| ≥ 2 and *p* < 0.05, of which 1471 mRNAs were upregulated and 792 mRNAs were downregulated (Supplementary Data S6). The hierarchical clustering also showed distinguishable expression levels of these differentially expressed mRNAs (Figure 3C).

Moreover, functional annotation of differential expressed mRNAs were also performed by GO and KEGG pathway analyses (Supplementary Datas S7, S8). The top five enriched GO terms in different domains for differential expressed mRNAs were showed in Figure 3D, and as indicated in Figure 3E, the top three enriched KEGG pathways are “Neuroactive ligand-receptor interaction”, “Cytokine-cytokine receptor interaction”, “TNF signaling pathway”, respectively.

### Validation of randomly selected circular ribonucleic acids and messenger RNAs

We randomly selected eight circRNAs and eight mRNAs to verify the circRNA and mRNA expression profiles. qRT-PCR analysis was performed on samples from the Sham and LSRA groups, and the expression levels of circRNAs and mRNAs were normalized to Gapdh expression levels.

**FIGURE 4:**
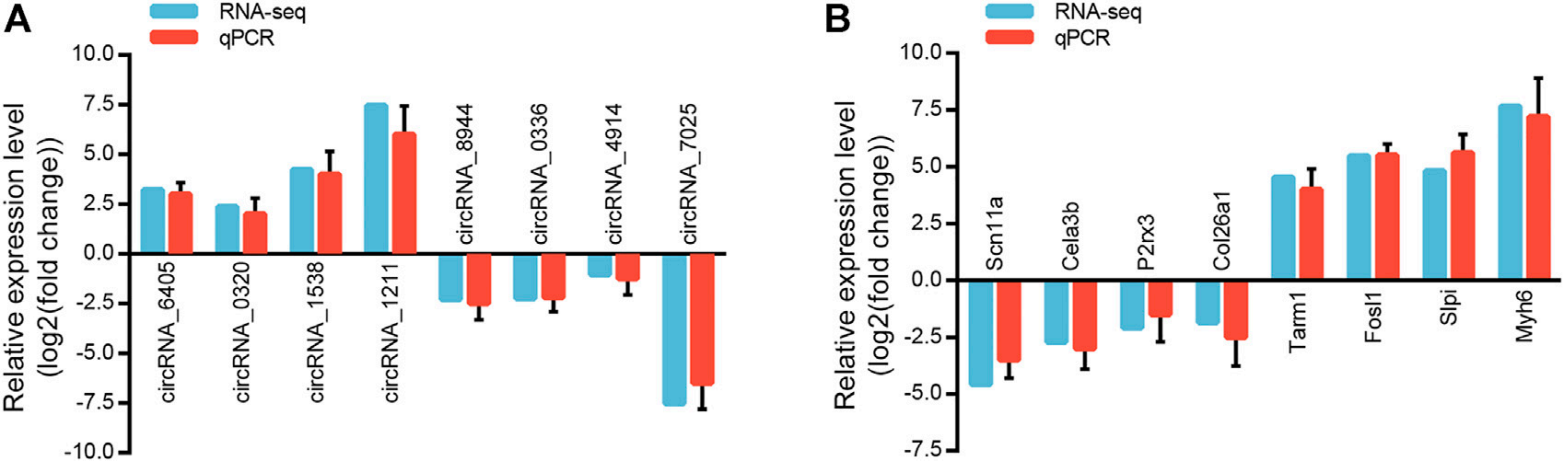
Validation of randomly selected circRNAs and mRNAs. **(A)** qRT-PCR analysis of eight randomly selected circRNAs (*n* = 3 for RNA-seq; *n* = 10 for qRT-PCR). The Y-axis represents the ratio of the relative expression of circRNAs in the LSRA group to that of the Sham group. **(B)** qRT-PCR analysis of eight randomly selected mRNAs (*n* = 3 for RNA-seq; *n* = 10 for qRT-PCR). The Y-axis represents the ratio of the relative expression of mRNAs in the LSRA group to that of the Sham group. circRNAs, circular RNAs; mRNAs, messenger RNAs; qRT-PCR, quantitative real-time polymerase chain reaction; LSRA, lumbosacral spinal root avulsion.

As shown in Figure 4A, the expression of circRNA_6405, circRNA_0320, circRNA_1538 and circRNA_1211 were markedly upregulated in the LSRA group, while the expression of circRNA_8944, circRNA_0336, circRNA_4914 and circRNA_7025 were significantly downregulated. Likewise, the expression levels of the eight randomly selected mRNAs are also in accordance with the RNA-seq results (Figure 4B). These data indicates the high credibility of our RNA-seq profiles.

### Construction of the circular ribonucleic acid/microRNA/messenger RNA networks

**FIGURE 5:**
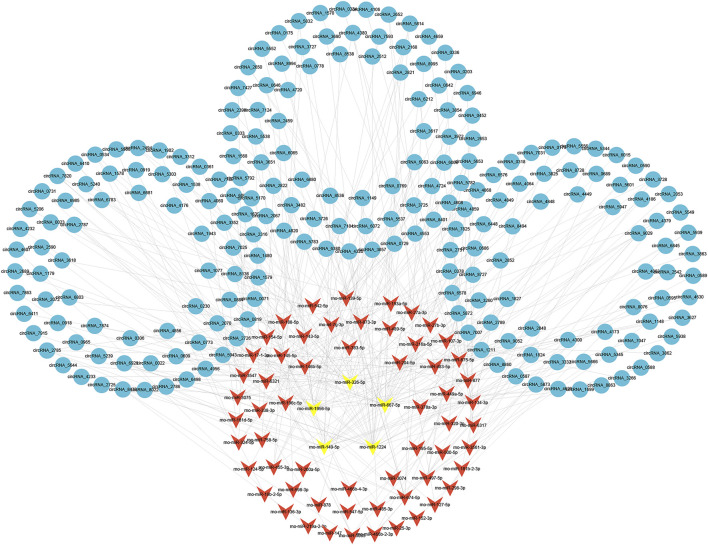
Construction of circRNA-miRNA network. The circRNA-miRNA interaction network for differentially expressed circRNAs. The top five miRNAs targeted by a larger number of circRNAs are marked in yellow. circRNAs, circular RNAs; miRNAs, microRNAs.

To evaluate the potential functions of circRNAs, we then predicted targeted miRNAs of differentially expressed circRNAs through the miRanda databases. Cytoscape was used to construct a circRNA/miRNA network to illustrate the top 300 circRNA-miRNA interactions ranked by *p* value of the hypergeometric distribution (Supplementary Data S9). In all, 211 circRNAs and 65 miRNAs were showed in the network, and among these miRNAs, five miRNAs (rno-miR-1224, rno-miR-667-5p, rno-miR-149-5p, rno-miR-1956, and rno-miR-326-5p) were proven to be regulated by a larger number of circRNAs than other miRNAs (Figure 5).

**FIGURE 6:**
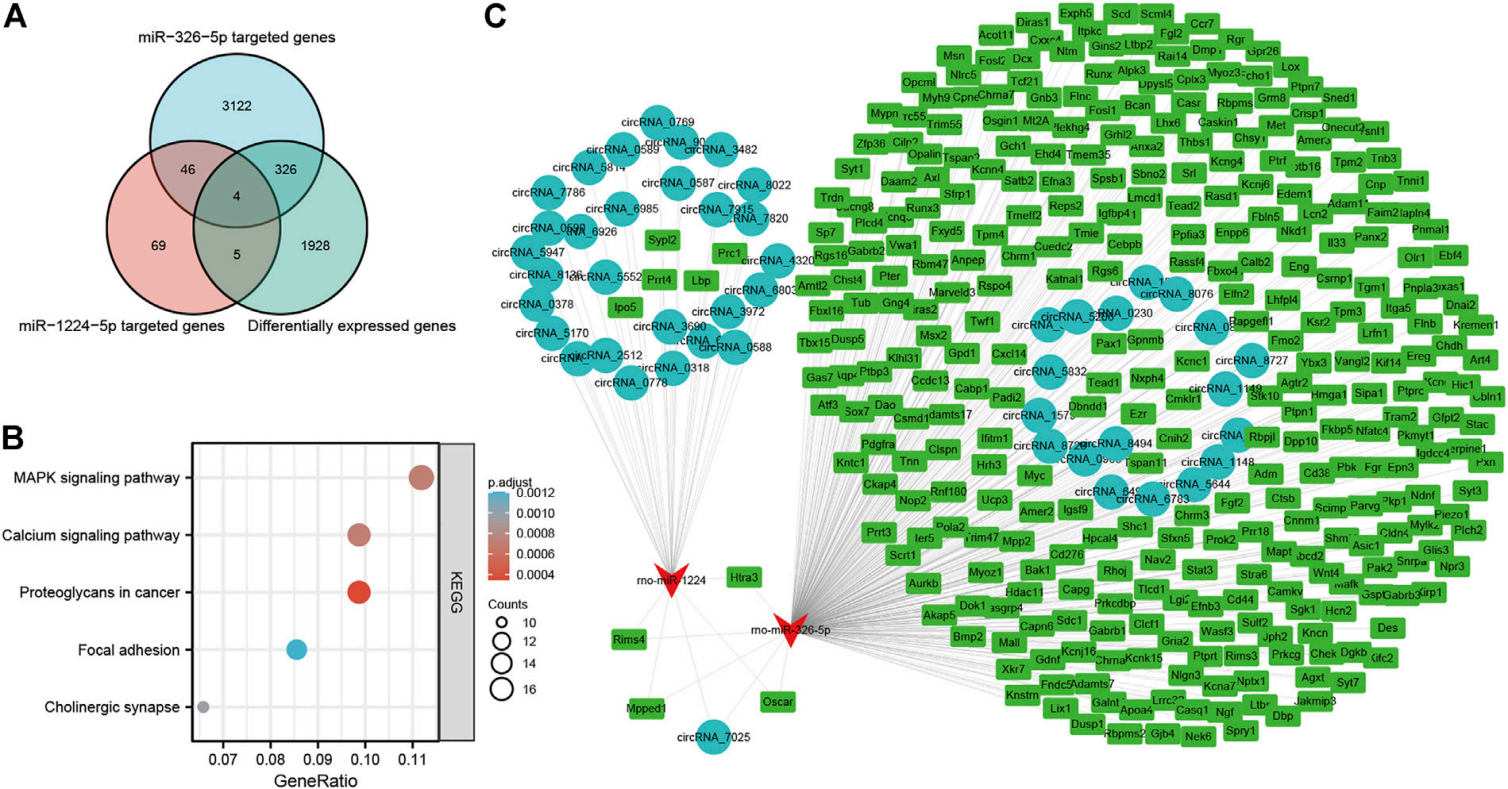
Construction of apoptosis-related circRNA-miRNA-mRNA network. **(A)** Venn diagram of differentially expressed mRNAs targeted by rno-miR-1224 and rno-miR-326-5p. **(B)** KEGG pathway analisis of differentially expressed mRNAs targeted by rno-miR-1224 and rno-miR-326-5p. **(C)** The apoptosis-related circRNA-miRNA-mRNAs interaction network of rno-miR-1224 and rno-miR-326-5p, and their targeted circRNAs and mRNAs. circRNAs, circular RNAs, miRNAs: microRNAs; mRNAs. messenger RNAs; KEGG, Kyoto Encyclopedia of Genes and Genomes.

Of the five miRNAs, rno-miR-1224 and rno-miR-326-5p were previously reported to be involved in neuronal apoptosis (Huang et al., 2021; Wan and Xiao, 2022), and our previous studies demonstrated that neuronal apoptosis might be a key physiological process in LSRA (Jiang et al., 2014; Zhou et al., 2019c). To construct a circRNA/miRNA/mRNA network related to neuronal apoptosis, we further predicted targeted mRNAs of rno-miR-1224 and rno-miR-326-5p, and intersected them with differentially expressed mRNAs identified by RNA-seq. As showed in Figure 6A, 335 differentially expressed mRNAs were found to be targeted by rno-miR-1224 or rno-miR-326-5p, and four of them (Oscar, Mpped1, Rims4, Htra3) were predicted to be targeted by both miRNAs (Supplementary Data S10). KEGG pathway analysis also revealed that these mRNAs were significantly enriched in “MARK signialing pathway” and “Calcium signaling pathway”, which were associated with apoptosis (Figure 6B). Then, 29 circRNAs targeting rno-miR-1224 and 19 circRNAs targeting rno-miR-326-5p were identified and a circRNA/miRNA/mRNA network was illustrated using Cytoscape software. Interestingly, circRNA_7025, a circRNA that was verified to be downregulated after LSRA, was predicted to be the target of both rno-miR-1224 and rno-miR-326-5p (Figure 6C).

### circRNA_7025 protects against neuronal apoptosis *via* the circRNA_7025- rno-miR-1224/rno-miR-326-5p axis

**FIGURE 7:**
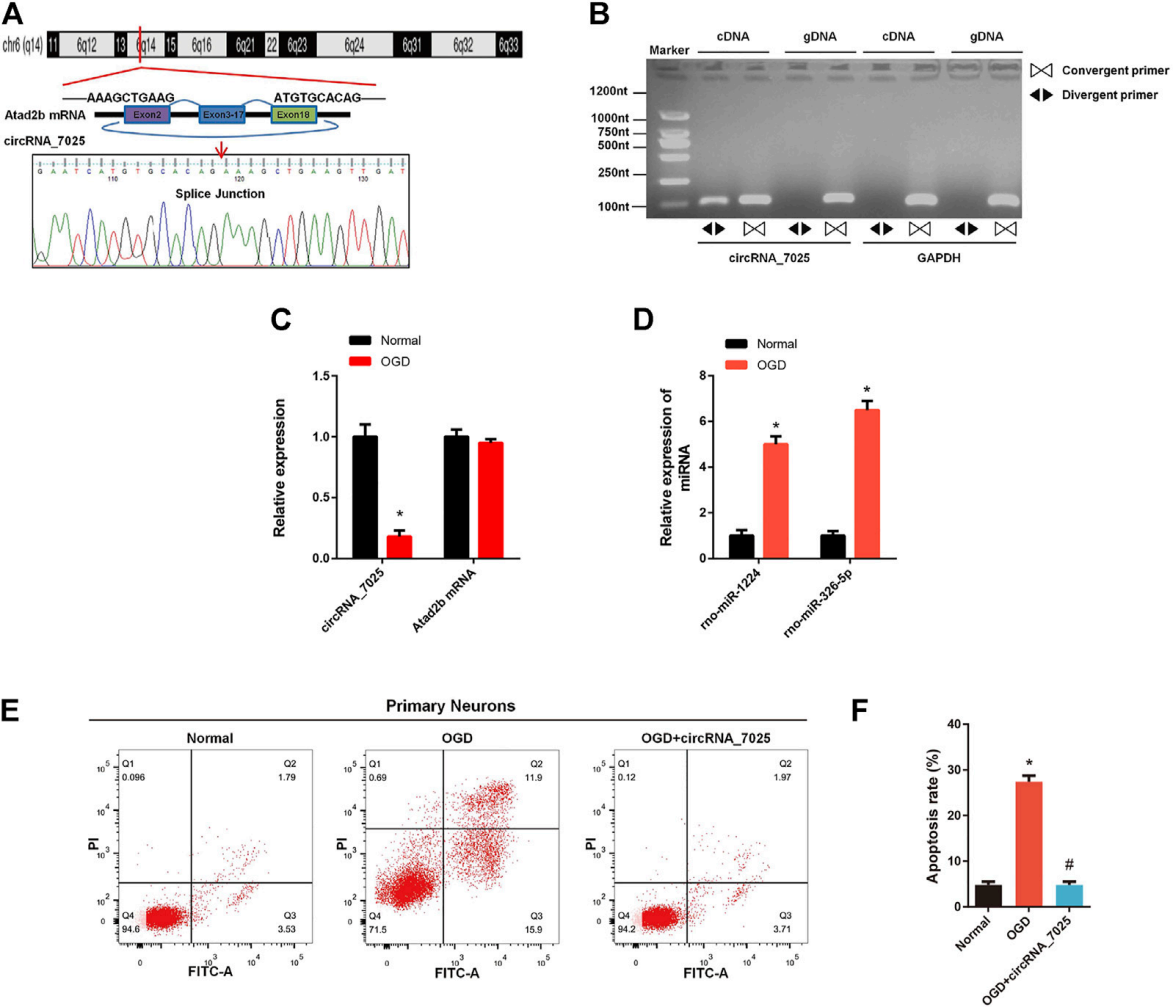
Effects of circRNA_7025 on OGD induced neuronal apoptosis. **(A)** Predicted splice junctions of circRNA_7025 were validated by Sanger sequencing. **(B)** PCR analysis for circRNA_7025 in cDNA and gDNA. Divergent primers amplified CircCDH13 from cDNA, but not from gDNA. **(C)** Relative expression level of circRNA_7025 and Atad2b mRNA after OGD treatment (*p < 0.05, *n* = 3). **(D)** Relative expression levels of rno-miR-1224 and rno-miR-326-5p in neurons after OGD treatment (*p < 0.05, *n* = 3). **(E)** The effect of circRNA_7025 on neuronal apoptosis was measured by flow cytometry. **(F)** The results of flow cytometry are presented as the apoptosis rate (*p < 0.05 vs. Normal group, #*p* < 0.05 vs. OGD group, *n* = 3). OGD, oxygen and glucose deprivation.

Furthermore, we found that circRNA_7025 was driven from exons 2 to 18 of the Atad2b gene on rat chr6, and the back-splice junction of circRNA_7025 was confirmed by Sanger sequencing (Figure 7A). We also designed specific convergent and divergent primers for circRNA_7025, and complementary DNA (cDNA) and genomic DNA (gDNA) were selected as the templates to perform PCR. Results showed that circRNA_7025 was amplified by divergent primers in cDNA but not in gDNA (Figure 7B), further confirming the existence of circRNA_7025. To reveal the potential role of circRNA_7025 in neuronal apoptosis, OGD treatment of primary spinal neurons was developed as an *in vitro* model to mimic the injured condition. The results showed that the expression of circRNA_7025 in primary neurons was downregulated after treatment of OGD, while the expression levels of Atad2b mRNA remained unchanged (Figure 7C). Furthermore, results also showed the expression of rno-miR-1224 and rno-miR-326-5p was upregulated OGD treated primary spinal neurons (Figure 7D). Then, an overexpression vector of circRNA_7025 was constructed to perform gain-of-function assays on neurons. As indicated in Figures 7E,F, apoptosis was induced in neurons after OGD treatment, and overexpression of circRNA_7025 markedly reduced the neuronal apoptosis rate, demonstrating an anti-apoptotic effect of circRNA_7025 in neurons.

**FIGURE 8:**
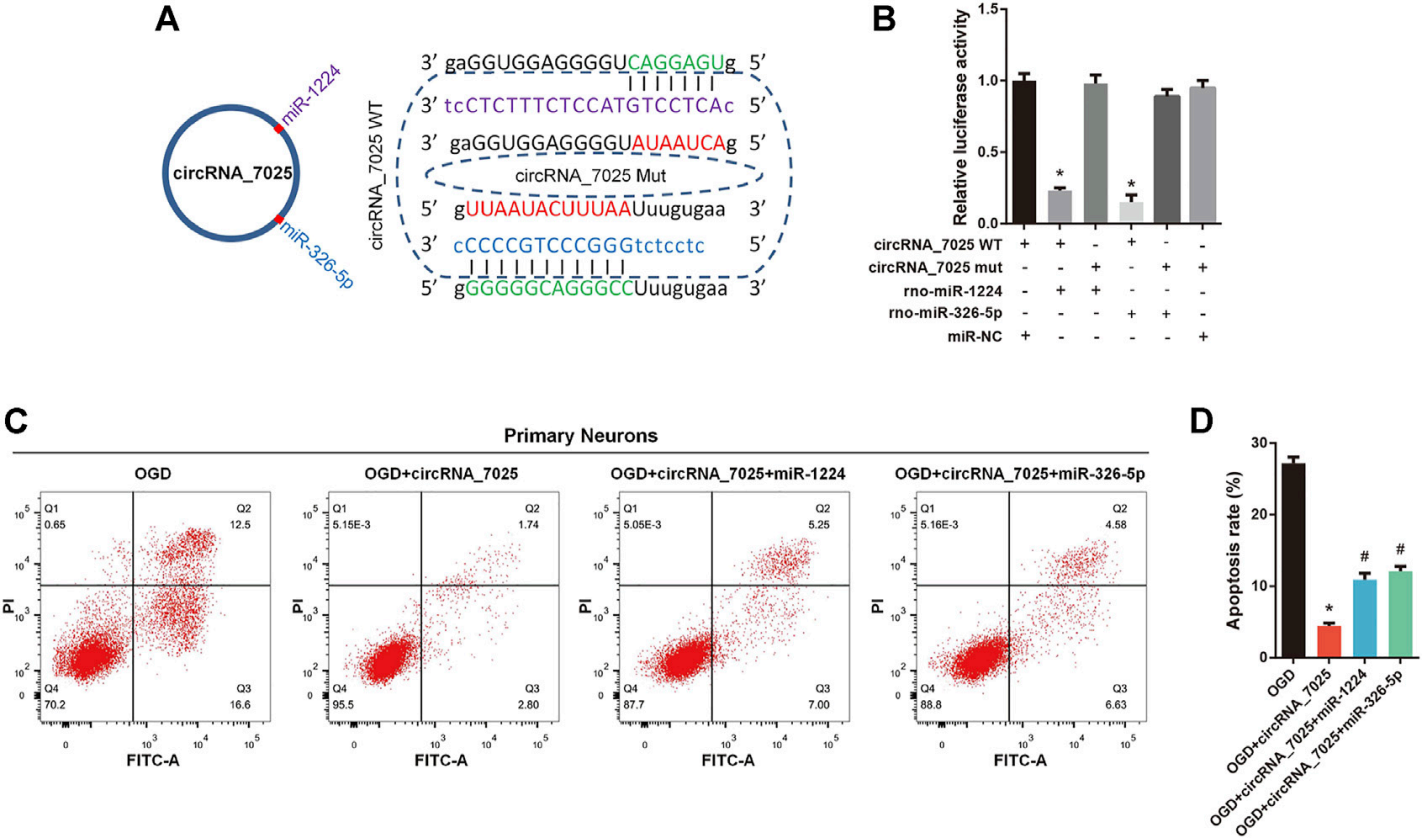
circRNA_7025 protects against neuronal apoptosis *via* the circRNA_7025- rno-miR-1224/rno-miR-326-5p axis. **(A)** Schematic diagrams of the predicted binding sites between circRNA_7025, rno-miR-1224 and rno-miR-326-5p, and construction of circRNA_7025 wild-type (WT) and mutant (Mut) luciferase reporter vectors. **(B)** Luciferase reporter assay for circRNA_7025 or circRNA_7025 mutant cotransfected with rno-miR-1224 and no-miR-326-5p mimics (*p < 0.05, *n* = 3). **(C)** Neuronal apoptosis was measured using flow cytometry. **(D)** The results of flow cytometry are presented as the apoptosis rate (*p < 0.05 vs. OGD group, #*p* < 0.05 vs. circRNA_7025 group, *n* = 3). OGD, oxygen and glucose deprivation.

Then, we focused on rno-miR-1224 and rno-miR-326-5p, which have been reported to be associated with neuronal apoptosis. We found that both rno-miR-1224 and rno-miR-326-5p have a complementary binding site for circRNA_7025 (Figure 8A). The luciferase assay showed a significant reduction in luciferase intensity when mimics of rno-miR-1224 and rno-miR-326-5p were cotransfected with circRNA_7025 wild-type luciferase reporter vectors, while there were no change in luciferase intensity when the binding sites of rno-miR-1224 and rno-miR-326-5p in circRNA_7025 were mutated, confirming the binding relationship between circRNA_7025 and rno-miR-1224 and rno-miR-326-5p (Figure 8B). Moreover, we studied whether circRNA_7025 exerts a protective effect in neurons *via* interacting with rno-miR-1224 and rno-miR-326-5p. Flow cytometry showed that the inhibition of neuronal apoptosis after circRNA_7025 overexpression was partly suppressed by rno-miR-1224 and rno-miR-326-5p mimics (Figures 8C,D). Together, these results suggest that circRNA_7025 protects against neuronal apoptosis *via* the circRNA_7025- rno-miR-1224/rno-miR-326-5p axis.

## Discussion

Lumbosacral spinal root avulsion is usually caused by high-energy trauma such as traffic accidents and high falls, which causes a progressive loss of neurons in the corresponding spinal cord, and is currently considered to be the most challenging to treat with the most devastating outcomes among nerve injuries (Sidhu and Dhillon, 1991). It causes permanent disabilities including motor impairment and sensory deficits in the lower limbs, as well as bowel, bladder and sexual dysfunctions (Chin and Chew, 1997). Although LSRA has been studied for more than 50 years, the functional recovery is far from optimal (Lang et al., 2004). The main reason is the involvement of complex molecular and biological changes in LSRA, and we lack sufficient understanding of the pathophysiology process.

Due to the development of new sequencing methodologies and bioinformatic tools, circRNAs are becoming the focus of RNA study. Recent studies have shown that circRNAs are a novel heterogeneous class of noncoding RNAs that are widely involved in various forms of diseases, including peripheral and central nerve injury (Xie et al., 2018; Zhao et al., 2018; Zhou et al., 2018; Zhou et al., 2019b). However, there has been no report focused on the function of circRNAs in LSRA. In the present study, we explored the circRNAs expression profiles in the corresponding spinal cord tissues after LSRA and found a group of differentially expressed circRNAs and mRNAs. Then, qRT-PCR revealed that the expression levels of randomly selected circRNAs and mRNAs were in accordance with the sequencing results, thus confirming the credibility of the RNA-seq data. To date, our study is the first one to revealing the expression profiles of circRNAs in the process of LSRA.

To predict the potential biological functions of differentially expressed circRNAs, the GO and KEGG pathway analyses were performed. GO enrichment analysis revealed multiple biological functions, which are associated with the physiological functions of neurons. KEGG analysis showed significant enrichment of differentially expressed circRNAs in pathways related to neuronal injury, such as the “MAPK signaling pathway” and “cAMP signaling pathway”. For example, previous evidence has shown that the MAPK signaling pathway plays a vital role in spinal cord injury and ischemic stroke (Tian et al., 2020; Zhang et al., 2021), while the cAMP signaling pathway is a critical regulator of neuronal apoptosis (Liu et al., 2020; Yu et al., 2022). Besides that, functional annotation also showed that the top five differentially expressed circRNAs were enriched in distinct GO and KEGG pathways, demonstrating important roles of these circRNAs may play in LSRA. These data preliminarily explored the potential functions of differentially expressed circRNAs identified by RNA-seq and proved that they are worthy of further investigation.

Emerging evidence has confirmed that circRNAs can act as competing RNAs of miRNAs and subsequently modulate their downstream functions. For example, circRNA HRCR was reported to be a sponge of miR-223 and protects the heart against hypertrophy and heart failure (Wang et al., 2016). CircPSMC3 contributes to the progression of gastric cancer by sponging miRNA-296-5p (Rong et al., 2019). Circ-MALAT1 functions as a microRNA sponge to promote self-renewal of hepatocellular cancer stem cells (Chen et al., 2020). To determine the potential functions of differentially expressed circRNAs, we also constructed a circRNA-miRNA network, and an interaction network of 211 circRNAs and 65 miRNAs was obtained, suggesting that these circRNAs can also function as sponges of miRNAs.

Previous studies showed that apoptosis was implicated in the pathophysiology of both central and peripheral nerve injury (Eldadah and Faden, 2000; Jia et al., 2020), and our previous work demonstrated that neuronal apoptosis was also induced in LSRA and might be a key factor restricting the surgical repair effects (Jiang et al., 2014; Zhou et al., 2019c). Specifically, among the predicted miRNAs, rno-miR-1224 and rno-miR-326-5p have been reported to be involved in neuronal apoptosis (Huang et al., 2021; Wan and Xiao, 2022), suggesting that they are likely to be involved in LSRA by regulating neuronal apoptosis. Moreover, we also identified differentially expressed mRNAs targeted by rno-miR-1224 and rno-miR-326-5p, and KEGG pathway analysis revealed that these mRNAs were also significantly enriched apoptosis-associated signaling pathway. Given that circRNAs can function as miRNA sponges and modulate gene expression by competitive binding with miRNAs (Memczak et al., 2013), a circRNA-miRNA-mRNA network related to neuronal apoptosis were constructed to further identified interacting relationships among circRNAs, miRNAs and mRNAs. Interestingly, circRNA_7025, a circRNA that was verified to be downregulated after LSRA, was predicted to be the target of both rno-miR-1224 and rno-miR-326-5p, further indicating a potential role that circRNA_7025 plays in neuronal apoptosis.

OGD treatment is regarded as an *in vitro* model of nerve injury and is widely used for functional studies (Ryou and Mallet, 2018; Salvador et al., 2018). To further verify our hypothesis, gain-of-function and rescue-function experiments were performed on primary spinal neurons subjected to OGD. First, the existence of circRNA_7025 was confirmed by Sanger sequencing, and qRT-PCR results confirmed that the expression levels of circRNA_7025 were negatively correlated with the two miRNAs. Then, a luciferase assay confirmed the binding relationship between circRNA_7025 and rno-miR-1224, and rno-miR-326-5p. Finally, flow cytometry showed that neuronal apoptosis induced by OGD was markedly reduced by overexpression of circRNA_7025, while this effect was partly suppressed by rno-miR-1224 and rno-miR-326-5p treatment. All the data suggests that the circRNA_7025 protects against neuronal apoptosis *via* the circRNA_7025- rno-miR-1224/rno-miR-326-5p axis.

This is the first study to characterize the circRNA expression profiles and explore their potential functions and mechanisms in LSRA. However, there are several limitations in this study. First, our results explored the differentially expressed circular RNAs of the samples after LSRA for 1 d, and further studies are needed on time-dependent changes in circRNAs and the precise mechanisms of molecular regulation of circRNAs. Second, we found that circRNA_7025 might protect against neuronal apoptosis *via* the circRNA_7025- rno-miR-1224/rno-miR-326-5p axis by bioinformatic analysis and *in vitro* experiments. Further *in vivo* studies will be needed to verify our results, and investigations should be conducted on other differentially expressed circRNAs and their regulatory mechanisms. Third, although rats share a high degree of sequence homology with humans, all the data obtained in this study were based on rats, further studies are needed to determine if these results can be successfully applied to humans.

In conclusion, base on bioinformatic analysis and circRNA/miRNA/mRNA interaction networks, our study identified that circRNA alterations were involved in the LSRA model of rats and predicted their potential functions. In addition, we also confirmed that circRNA_7025, a downregulated circRNA, plays an anti-apoptotic role in LSRA *via* the circRNA_7025/rno-miR-1224/rno-miR-326-5p axis. The findings improve our understanding of the pathogenic mechanisms and enable us to identify new molecular targets for treating neuronal apoptosis after LSRA.

## Data Availability

The data presented in the study are deposited in the Gene Expression Omnibus (GEO), accession number GSE203053.
