# Supplementary material for: Bioinformatic analysis of circular RNA expression profiles in a rat lumbosacral spinal root avulsion model
Source: Front Genet. 2022 Aug 12;13:920493. doi: 10.3389/fgene.2022.920493 (PMC9412201; doi:10.3389/fgene.2022.920493)
Supplement: Supplementary file 3 [file Table1.DOCX]

## Supplementary Data 1 Primers used for qRT-PCR.

| **circRNA** | **Forward primer** | **Reverse primer** |
| --- | --- | --- |
| **circRNA_1211** | CGGGAAGACTGTGAACACGA | CACAGATTCGGGCCAATGGA |
| **circRNA_1538** | TCCCAGGTTCAAGCCTTTGG | ACAGGGGCAACTCCAGAAAC |
| **circRNA_6405** | TGAATTGGCGGCTTTGTTCT | CACCGTAATACACGCTGTGC |
| **circRNA_0320** | TTGGGAGAGATGAACGAGGAG | TAGCTTCAGCACCATCTTGGA |
| **circRNA_4914** | ACGGAGAGACCTGGAAGGAA | TGTCTGCTGCCCAACTGTAG |
| **circRNA_0336** | AGCCATGCTTTCTCTCAGCA | AGCACATGGGCACATCGTTA |
| **circRNA_8944** | TTCTGCTTACACCACCAGGC | GATGAAGTCAGAGAGGCCCG |
| **circRNA_7025** | GGGAACGAGGTTCAGGACAG | GTTTCAAAGTGCGCTTGGCT |
| **rno-miR-326-5p** | CAGGGCCTTTGTGAA | TCAACTGGTGTCGTGG |
| **rno-miR-1224** | GTGAGGACTCGGGAGGTGG | AGCTCAGACCCCTTTACCAC |
| **Scn11a** | GAAAAAGTTAGGTGGCCAAGACAT | GTTGGGCTGGCCTTCAGATT |
| **Cela3b** | TCATGGGCCTCAGAAACACT | TGTTGTTCTTGCTGGTGTCG |
| **P2rx3** | TGGACAGGATTTTGCCAAGC | CTGAAGTTGTAGCCAGGGGA |
| **Col26a1** | CTGTCAACTTTGTGTCCCGG | TGGTGTTGTTGTTGTTGGCA |
| **Tarm1** | ATGATCCTCGTCTACCTGCG | CAGGATCTTCAGGGGTCTCC |
| **Fosl1** | ATGTTTTGTGAGCTGCCCTG | CATGCCCAGGTCAAAGATCG |
| **Slpi** | TTCTCTCCAGCCCTTTCCAG | AAAGCGGTCTGACTCCTTGA |
| **Myh6** | GTGGGCAAGTGTCATCGTTT | AGACATCCTGAGAGAGTGCG |
